# Supplementary material for: The vaginal metabolomics profile with features of polycystic ovary syndrome: a pilot investigation in China
Source: PeerJ. 2024 Oct 8;12:e18194. doi: 10.7717/peerj.18194 (PMC11468964; doi:10.7717/peerj.18194)
Supplement: Supplemental Information 2 — Note: a Pearson correlations were used. Spearman correlations were used. Abbreviations: BMI, body mass index. [file peerj-12-18194-s002.docx]

**Table S1** Correlation analysis of differential metabolites and sociodemographic characteristics and health indicators

| **Metabolites** | **Trend** | **Statistic** | **Age^a^** | **Occupation^b^** | **Educational level^b^** | **BMI^a^** | **Fasting plasma glucose^a^** | **Menstruation period^b^** | **Dysmenorrhea^b^** | **Vaginal cleanness^b^** | **Vaginal pH^a^** | **Bacterial vaginosis infection^b^** |
| --- | --- | --- | --- | --- | --- | --- | --- | --- | --- | --- | --- | --- |
| Bovinic acid | ↑ | *r* | -0.55 | -0.36 | 0.08 | -0.06 | 0.26 | 0.85 | 0.37 | 0.37 | 0.21 | 0.32 |
|  |  | *P* | 0.01 | 0.12 | 0.72 | 0.81 | 0.27 | <0.001 | 0.11 | 0.11 | 0.37 | 0.17 |
| Dopamine | ↑ | *r* | -0.30 | -0.56 | 0.06 | 0.19 | -0.09 | 0.79 | 0.49 | 0.43 | 0.55 | 0.23 |
|  |  | *P* | 0.20 | 0.01 | 0.80 | 0.43 | 0.72 | <0.001 | 0.03 | 0.06 | 0.01 | 0.33 |
| Gentisic acid | ↑ | *r* | -0.09 | -0.26 | 0.18 | -0.15 | 0.36 | 0.85 | 0.40 | 0.44 | 0.32 | 0.32 |
|  |  | *P* | 0.71 | 0.28 | 0.44 | 0.53 | 0.12 | <0.001 | 0.08 | 0.05 | 0.17 | 0.17 |
| Homovanillic acid | ↑ | *r* | -0.26 | -0.35 | -0.11 | 0.12 | -0.07 | 0.83 | 0.27 | 0.28 | 0.71 | 0.32 |
|  |  | *P* | 0.26 | 0.13 | 0.65 | 0.63 | 0.78 | <0.001 | 0.25 | 0.23 | <0.001 | 0.17 |
| Homovanillin | ↑ | *r* | -0.01 | -0.58 | 0.26 | -0.14 | 0.14 | 0.83 | 0.21 | 0.39 | 0.45 | 0.26 |
|  |  | *P* | 0.99 | 0.01 | 0.27 | 0.57 | 0.55 | <0.001 | 0.37 | 0.09 | 0.05 | 0.26 |
| Hydroxyphenyllactic acid | ↑ | *r* | 0.26 | -0.25 | 0.28 | 0.08 | 0.36 | 0.81 | 0.26 | 0.34 | 0.64 | 0.40 |
|  |  | *P* | 0.27 | 0.30 | 0.23 | 0.75 | 0.12 | <0.001 | 0.27 | 0.15 | <0.001 | 0.08 |
| Linoleic acid | ↑ | *r* | -0.43 | -0.14 | -0.13 | -0.09 | 0.02 | 0.65 | 0.38 | 0.37 | 0.29 | 0.2 |
|  |  | *P* | 0.06 | 0.56 | 0.57 | 0.72 | 0.95 | 0.01 | 0.10 | 0.11 | 0.22 | 0.39 |
| m-Coumaric acid | ↑ | *r* | -0.20 | -0.23 | 0.13 | 0.19 | 0.18 | 0.83 | 0.34 | 0.21 | 0.76 | 0.12 |
|  |  | *P* | 0.39 | 0.32 | 0.57 | 0.42 | 0.46 | <0.001 | 0.15 | 0.37 | <0.001 | 0.63 |
| N1-Methyl-2-pyridone-5-carboxamide | ↑ | *r* | -0.32 | -0.56 | 0.23 | -0.07 | 0.10 | 0.79 | 0.37 | 0.50 | 0.41 | 0.26 |
|  |  | *P* | 0.17 | 0.01 | 0.33 | 0.77 | 0.97 | <0.001 | 0.11 | 0.03 | 0.07 | 0.27 |
| Niacinamide | ↑ | *r* | 0.19 | -0.25 | 0.13 | -0.03 | 0.26 | 0.67 | 0.19 | 0.41 | 0.39 | 0.40 |
|  |  | *P* | 0.42 | 0.28 | 0.57 | 0.89 | 0.27 | 0.01 | 0.43 | 0.08 | 0.09 | 0.08 |
| Phenylacetic acid | ↑ | *r* | -0.29 | -0.42 | 0.11 | 0.01 | 0.10 | 0.86 | 0.39 | 0.50 | 0.25 | 0.38 |
|  |  | *P* | 0.21 | 0.07 | 0.65 | 0.99 | 0.69 | <0.001 | 0.09 | 0.03 | 0.29 | 0.10 |
| p-Hydroxyphenylacetic acid | ↓ | *r* | 0.15 | 0.42 | -0.06 | -0.23 | 0.05 | -0.83 | -0.30 | -0.23 | -0.69 | -0.23 |
|  |  | *P* | 0.52 | 0.06 | 0.80 | 0.33 | 0.84 | <0.001 | 0.19 | 0.33 | <0.001 | 0.33 |
| Prostaglandin G2 | ↓ | *r* | 0.25 | 0.41 | -0.18 | -0.27 | -0.08 | -0.82 | -0.54 | -0.27 | -0.70 | -0.36 |
|  |  | *P* | 0.30 | 0.07 | 0.46 | 0.25 | 0.75 | <0.001 | 0.01 | 0.24 | <0.001 | 0.12 |
| Trigonelline | ↑ | *r* | -0.28 | -0.22 | -0.01 | 0.38 | 0.26 | 0.78 | 0.42 | 0.18 | 0.53 | 0.43 |
|  |  | *P* | 0.24 | 0.34 | 0.96 | 0.10 | 0.27 | <0.001 | 0.06 | 0.46 | 0.02 | 0.06 |
| 3,4-Dihydroxyhydrocinnamic acid | ↑ | *r* | -0.30 | -0.33 | 0.18 | 0.13 | 0.12 | 0.79 | 0.44 | 0.41 | 0.36 | 0.38 |
|  |  | *P* | 0.19 | 0.15 | 0.44 | 0.59 | 0.60 | <0.001 | 0.05 | 0.08 | 0.12 | 0.10 |
| 3,4-Dihydroxymandelaldehyde | ↑ | *r* | -0.08 | -0.53 | 0.42 | -0.09 | 0.24 | 0.81 | 0.42 | 0.56 | 0.60 | 0.24 |
|  |  | *P* | 0.73 | 0.02 | 0.07 | 0.71 | 0.31 | <0.001 | 0.06 | 0.01 | 0.01 | 0.40 |
| 4-Hydroxybenzoic acid | ↑ | *r* | -0.56 | -0.24 | -0.08 | 0.01 | 0.08 | 0.83 | 0.29 | 0.28 | 0.14 | 0.38 |
|  |  | *P* | 0.01 | 0.31 | 0.72 | 0.99 | 0.75 | <0.001 | 0.22 | 0.03 | 0.56 | 0.10 |
| 11-Dehydro-thromboxane B2 | ↓ | *r* | 0.16 | 0.46 | -0.13 | -0.28 | 0.10 | -0.75 | -0.41 | -0.26 | -0.68 | -0.36 |
|  |  | *P* | 0.50 | 0.04 | 0.60 | 0.24 | 0.67 | <0.001 | 0.07 | 0.28 | <0.001 | 0.12 |

Note: ^a^ Pearson correlations were used. ^b^ Spearman correlations were used. **Abbreviations:** BMI = body mass index.
