# Supplementary material for: The vaginal metabolomics profile with features of polycystic ovary syndrome: a pilot investigation in China
Source: PeerJ. 2024 Oct 8;12:e18194. doi: 10.7717/peerj.18194 (PMC11468964; doi:10.7717/peerj.18194)
Supplement: Supplemental Information 1 — (A) PCA score plot in positive ion mode. (B) PCA score plot in negative ion mode. Abbreviations: PCA, principal component analysis; PCOS, polycystic ovary syndrome; QC, quality control. [file peerj-12-18194-s001.pdf]

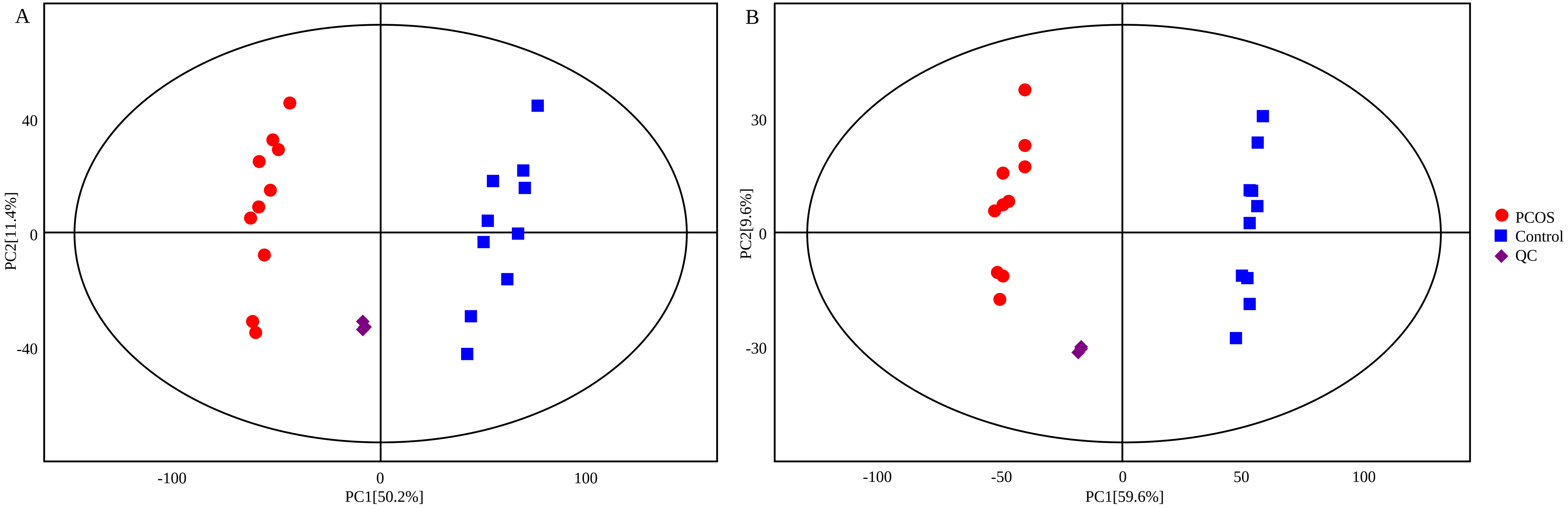
A
40
0
-40
-100
0
100
PC1[50.2%]
PC2[11.4%]
B
30
0
-30
-100
-50
0
50
100
PC1[59.6%]
PC2[9.6%]
PCOS
Control
QC
